# Balancing STAT Activity as a Therapeutic Strategy

**DOI:** 10.3390/cancers11111716

**Published:** 2019-11-03

**Authors:** Kelsey L. Polak, Noah M. Chernosky, Jacob M. Smigiel, Ilaria Tamagno, Mark W. Jackson

**Affiliations:** 1 Department of Pathology, Case Western Reserve University, School of Medicine, Cleveland, OH 44106, USA; kxp328@case.edu (K.L.P.); nmc71@case.edu (N.M.C.); jxs1094@case.edu (J.M.S.); ixt64@case.edu (I.T.); 2 Case Comprehensive Cancer Center, Case Western Reserve University, School of Medicine, Cleveland, OH 44106, USA

**Keywords:** STAT3, STAT5, cancer progression, cancer-stem cell, cytokine, therapy resistance, metastasis, immunosuppression, tumor microenvironment, proliferation

## Abstract

Driven by dysregulated IL-6 family member cytokine signaling in the tumor microenvironment (TME), aberrant signal transducer and activator of transcription (STAT3) and (STAT5) activation have been identified as key contributors to tumorigenesis. Following transformation, persistent STAT3 activation drives the emergence of mesenchymal/cancer-stem cell (CSC) properties, important determinants of metastatic potential and therapy failure. Moreover, STAT3 signaling within tumor-associated macrophages and neutrophils drives secretion of factors that facilitate metastasis and suppress immune cell function. Persistent STAT5 activation is responsible for cancer cell maintenance through suppression of apoptosis and tumor suppressor signaling. Furthermore, STAT5-mediated CD4+/CD25+ regulatory T cells (T_regs_) have been implicated in suppression of immunosurveillance. We discuss these roles for STAT3 and STAT5, and weigh the attractiveness of different modes of targeting each cancer therapy. Moreover, we discuss how anti-tumorigenic STATs, including STAT1 and STAT2, may be leveraged to suppress the pro-tumorigenic functions of STAT3/STAT5 signaling.

## 1. Introduction

A complex milieu of both cellular and non-cellular components creates a heterogeneous tumor and microenvironment [1,2,3,4,5,6,7,8]. As a tumor becomes more heterogeneous, the risk of metastasis and therapy failure increases [9,10,11,12]. Cancer cells, pericytes, immune cells, fibroblasts, and endothelial cells are just some of the cellular components of the tumor [13,14]. A highly dysregulated network of cytokines and growth factors, emanating from cancer and stromal tumor microenvironment (TME) cells, contributes to the evolution of cancer cells, and often, suppression of immune cell function. Many of these cytokines and growth factors result in the phosphorylation and activation of signal transducers and activators of transcription (STAT) proteins, which can drive cell-specific changes in gene expression. STAT3 and STAT5 activity is often elevated in aggressive subtypes of cancer and serve as prognostic indicators [15,16,17,18,19,20,21,22,23,24]. Here, we discuss the impact of microenvironmental signals on STAT3/STAT5 activation during cancer development and progression. Elevated STAT3 activity promotes epithelial-mesenchymal transition (EMT) and a stem cell program in cancer cells, while also suppressing the function of immune cells within the tumor, all of which are important steps that underlie metastasis and therapy failure [25,26,27,28,29,30]. Likewise, persistent STAT5 activity induces (i) transformation, (ii) proliferation, and (iii) anti-apoptotic signals that contribute to hematological malignancies, while also suppressing anti-tumor immunity by expanding CD4+/CD25+ regulatory T cells (T_regs)_ [28,31,32,33,34,35,36]. We will discuss options for targeting STAT3/5 in cancer, either directly or indirectly through the inhibition of upstream kinases, receptors and/or ligands. In addition, we will discuss how STAT1/2 activity can counter the more aggressive phenotypes induced by STAT3/5. We propose that balancing STAT-activated cytokine signaling in the TME may serve as an effective therapeutic strategy.

## 2. Activating STAT3 and STAT5

**Figure 1:**
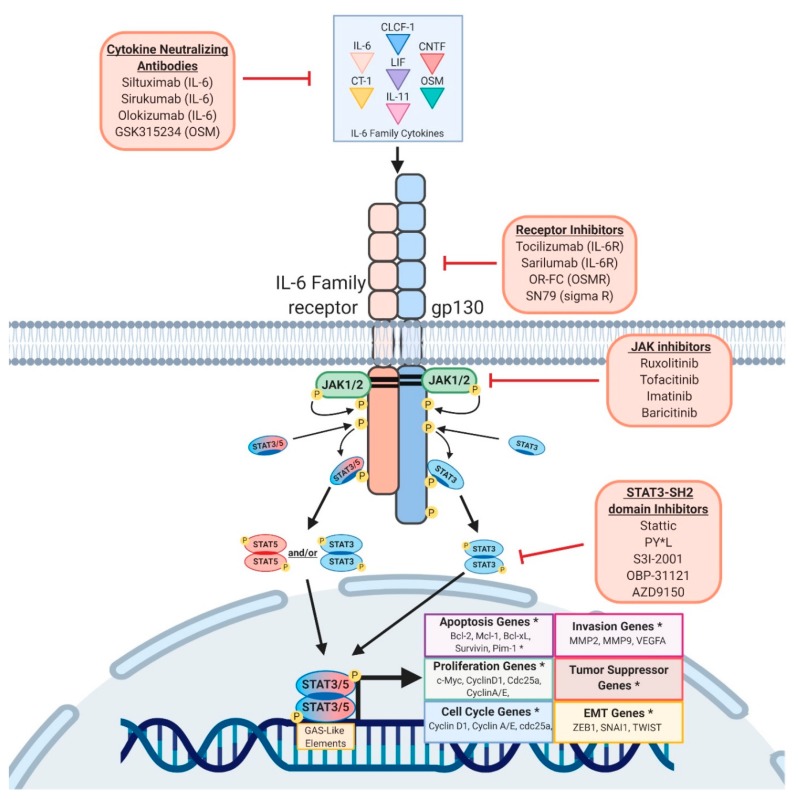
STAT3/5 Signaling Cascades and Therapy Targets. Schematic representation of canonical activation of signal transducer and activator of transcription-3 (STAT3) and STAT5 by IL-6 family member cytokines. IL-6 family cytokines drive receptor heterodimerization and subsequent Janus Kinase (JAK activation). JAKs phosphorylate tyrosine residues along the cytoplasmic domain of the receptor dimer, which recruits STAT proteins and facilitates their binding to interferon-gamma activation site-like (GAS-like) elements and regulation of large sets of genes. Asterisks (*) denote that additional info can be found in Table 1.

The STAT family of transcription factors is comprised of seven different members, STAT1, -2, -3, -4, -5a, -5b, and -6. Hereafter, when discussing the overlapping functions of STAT5A and STAT5B, we will refer to them as “STAT5”. STAT proteins transduce signals from the cell membrane to the nucleus, bypassing the need for second messengers [37,38,39]. STATs are phosphorylated by Janus Kinases (JAK1, 2, 3) or Tyrosine Kinase 2 (Tyk2), which are recruited to ligand-activated receptors, including cytokine, growth factor, or g-protein associated receptors. Most important of the STAT3/5 activators are the IL-6 family members, which include IL-6, IL-11, ciliary neurotrophic factor (CNTF), leukemia inhibitory factor (LIF), oncostatin-m (OSM), cardiotrophin 1 (CT-1), cardiotrophin-like cytokine (CLC), and IL-27. IL-6 family cytokines form a heterodimeric complex consisting of gp130 and a cytokine specific subunit (IL-6Rα, IL-11Rα, CNTFRα, gp130, LIFR, and OSMR), recruiting and activating the JAKs or Tyk2. Once phosphorylated, STATs dimerize and translocate to the nucleus, where they bind to short stretches of DNA and act as transcription factors to induce the expression of genes implicated in cell proliferation, survival, differentiation, motility, apoptosis, and metabolism (Figure 1) [37,40,41,42,43].

The crucial role STATs play in these normal physiological processes was first demonstrated in STAT-deficient mouse models. The generation of tissue-specific STAT3 knockout models have identified STAT3 as a key component in a wide variety of processes, including, but not limited to: T-cell proliferation, suppression of apoptosis, epidermal regeneration during wound healing, macrophage and neutrophil anti-inflammatory responses, and mammary gland involution [44,45,46]. Deletion of STAT5 in mice demonstrated high incidence of perinatal lethality, prevented the appropriate development of B-cells and T-cells, and inhibited the function of hematopoietic stem cells [47]. Under normal conditions, JAK/STAT activation is tightly regulated by protein inhibitors of activated STATs (PIAS), tyrosine phosphatases, and suppressors of cytokine signaling (SOCS) proteins that inhibit JAK catalytic activity [48,49]. In cancer however, STAT3 and STAT5 activity becomes dysregulated, resulting in elevated STAT3/5-driven responses in tumor, stromal, and immune cells.

Advances in sequencing technologies have allowed scientists to investigate the frequency of mutations in the JAK/STAT signaling pathway, resulting in the identification of mutations that constitutively activate STAT3, STAT5, and JAK2. The majority of STAT mutations occur in the SH2 and C-terminal domains of STAT3 and STAT5B and associate with leukemias and lymphomas [50,51]. STAT3 is the most frequently mutated member of the STAT family, with high incidence of mutation in T-cell large granular lymphocytic leukemias and NK lymphoproliferative disorders [52,53]. STAT5B mutations were similarly identified in these diseases, but with lower frequency. In addition, sequencing of STAT3 exons in diffuse large B-cell lymphoma patients identified a missense point mutation (M206K) in the coiled-coil domain that affected SH2 domain function and drove robust proliferation [54,55]. Furthermore, JAK2 V617F, a constitutively active JAK2 mutant, phosphorylates both STAT3 and STAT5 proteins and has high frequency in patients with hematopoietic stem cell diseases, such as myeloproliferative diseases, essential thrombocythemia, polycythemia vera (PV), and idiopathic myelofibrosis (IMF) [56]. In addition, JAK2 V617F mutations have transformation potential in in vivo bone marrow transplantation assays and induce persistent activation of STAT5 and a PV phenotype [57]. While persistent activation of both STAT3 and STAT5 has been implicated in the transformation process, the greater impact of dysregulated STAT3/5 appears to be their influence on the induction of aggressive cancer cell properties and immunosuppression. The impacts of both cell-intrinsic reprogramming and immune dysfunction are discussed below.

## 3. IL-6 Family Cytokine Dysregulation in the TME

A number of IL-6 family members have long been recognized for their involvement in the pathogenesis of aggressive cancers [19,77,78,79,80,81]. For example, IL-6 levels serve as a prognostic biomarker and predictor of therapeutic response in pancreatic ductal adenocarcinoma (PDAC), bladder, gastric, lung adenocarcinoma, colorectal, cervical, liver, and breast cancers: all of which have a high incidence of metastasis and resistance [77,82,83,84,85,86]. This is due to the pro-tumor effects of IL-6 in tumor cells and stromal components. In tumor cells, IL-6 can drive EMT, therapy resistance, and invasive characteristics [87,88,89]. Concurrently, IL-6 can shift the anti-tumor immune responses towards immunosuppression via recruitment of myeloid-derived suppressor cells (MDSCs) and expansion of FoxP3+ T_regs_ [90,91,92]. Furthermore, IL-6 secretion by certain stromal components (cancer-associated fibroblasts, macrophages, and neutrophils) drives EMT in associated tumor populations, which is supported by immunohistochemical staining at the invasive edge of patient breast tumors, where levels of phosphorylated STAT3 and IL-6 is elevated [93,94].

Interestingly, IL-6 activation of STAT3 functions in a positive feed-forward loop to drive the secretion of new IL-6 into the TME, which then interacts with IL-6R/gp130 to further activate JAK/STAT signaling [93,95]. OSM also potently activates a feed-forward loop, resulting in the de novo production of additional OSM and OSMR by tumor and immune cells [96]. Interestingly, OSM was first identified to play a tumor suppressive role and inhibited the proliferation of melanoma cell models [97]. However, later studies correlated OSM-OSMR signaling with robust STAT3 and STAT5 activation and more aggressive tumor phenotypes [98,99,100,101,102,103,104,105,106]. Like IL-6, OSM feed-forward signaling is observed in aggressive cancers with limited-therapeutic options, including glioblastoma (GBM), non-small cell lung carcinoma (NSCLC), PDAC, and triple negative breast cancer (TNBC) [107,108,109,110,111]. Elevated signaling through the OSM/OSMR axis induces high levels of an ‘inflammatory module’, which includes IL-6, CCL2, IL-1, CXCL1, CXCL9, CXCL10, and CXCL11—all of which have been implicated in migration, invasion, therapy failure, and dedifferentiation to a cancer stem cell (CSC) program. Importantly, neutralization of OSM by treatment with an Fc-tagged soluble OSMR suppresses this inflammatory module suggesting that the OSM/OSMR feed-forward loop may be critical for the long-term maintenance of inflammatory signaling [112,113].

IL-11 similarly engages in an autocrine feed-forward mechanism to drive persistent JAK2/STAT3 activation, which can promote resistance to platinum-based therapies [114]. Furthermore, IL-11 and LIF both contribute to tumorigenesis by enhancing tumor cell survival through STAT3-mediated activation of anti-apoptotic proteins, Bcl-2 and Survivin, and the inhibition of tumor suppressor p53 [114,115,116]. IL-11 and LIF also contribute to cancer progression by driving EMT through STAT3 and Akt/mTOR signaling, thereby conferring an enhanced migratory capacity [81,117,118]. The impact of CT-1, CLC, CNTF, and IL-27 on cancer progression is currently understudied. While IL-6 family cytokines are important mediators of STAT3 activation, other cancer-associated receptors can also activate STAT3. STAT3-driven tumor progression can also be achieved by epidermal growth factor receptors (both wild-type EGFR and EGFRvIII), fibroblast growth factor receptors (FGFR), insulin-like growth factor receptor (IGFR), hepatocyte growth factor (HGFR, also known as MET), platelet-derived growth factor receptor (PDGFR), vascular endothelial growth factor receptor (VEGFR), v-src, and Bcr-Abl [119,120,121,122,123,124,125,126,127]. Interestingly, acetylation of STAT3 also induces tumor progression through enhanced pro-tumorigenic IL-17A secretion [128,129]. These examples demonstrate the abundance of mechanisms through which STAT3 can become activated.

## 4. STAT3 Activation of a Mesenchymal/CSC Program in Cancer Cells

The majority of cancers have constitutive activation of STAT3 [130,131,132]. STAT3 was first termed oncogenic when its persistent activation was discovered in v-src transformed mouse embryonic fibroblasts [133]. Subsequent studies demonstrated that expression of a constitutively active STAT3 isoform (STAT3-C) can drive transformation of pre-malignant human mammary epithelial cells (HMEC) and MCF-10A cells to malignant breast cancer [134]. In addition, Ras-induced transformation in bladder and breast carcinoma models exhibit mitochondrial accumulation of STAT3 and more robust cellular glycolysis, a characteristic of cancer cells [135]. In PDAC, STAT3 is required for both the development of pre-malignant pancreatic interepithelial neoplasias (PanINs), as well as the progression of PanINs to PDAC [136]. While STAT3 has been strongly implicated as a driver of oncogenesis, evidence suggests that persistent cytokine activation of STAT3 in pre-malignant lesions, engages a tumor suppressive senescence response. In non-transformed HMEC models, OSM engages senescence through a direct STAT3 interaction with mothers against decapentaplegic-3 (SMAD3). However, downstream constitutive expression of c-Myc could overcome OSM-induced senescence and drive EMT and invasion [137]. Beyond the genetic events that occur as a normal cell becomes transformed, cancer progression relies heavily on an evolving microenvironment, which impacts both the cancer cells and the immune system, ultimately influencing patient outcomes. In cancer cells, increased STAT3 activity induces EMT-driving transcription factors, such as ZEB1, SNAIL, and Twist, which initiate the repression of epithelial markers and expression of mesenchymal markers (N-cadherin, Vimentin) [138,139,140,141]. In addition, STAT3 induces the expression of matrix metalloproteinases (MMP), which can also contribute to the loss of cell-cell contacts [142,143,144].

While EMT is important in normal physiology to allow cells to migrate during development and wound healing, inappropriate EMT and the loss of cell-cell interactions contributes to aggressive cancer cell behaviors, including metastasis and resistance to therapy. In TNBC, the loss of E-cadherin or gain of Vimentin, N-Cadherin, or Snail expression, typically at the tumor’s edge, correlates with poor clinical outcome [145,146,147,148,149]. Likewise, the presence and abundance of circulating tumor cells (CTC) that express mesenchymal markers have been referred to as the “silent predictors of metastasis” because they correlate with tumor cell dissemination [150]. Moreover, mesenchymal CTC can be used to track a patient’s response to therapy, with increasing numbers correlating with increased risk of relapse [151,152,153,154,155].

Likewise, the emergence of CSC properties, which are often defined experimentally as using tumorsphere- or tumor-initiating assays, frequently occur concomitantly with a mesenchymal phenotype [156,157,158,159,160,161,162,163]. STAT3 has been identified as an essential driver of the de novo reprogramming to a CSC state through activation of Sox2, Nanog, and Oct4 [164,165,166,167,168,169,170]. Further evidence linking EMT and a CSC phenotype is provided by the observation that a subset of CTC, responsible for initiating metastasis termed the metastasis-initiating cells, express high levels of the STAT3-driven CSC marker CD44 [151,152,153,171,172,173,174]

The importance of mesenchymal/CSC reprogramming in metastasis and therapy failure continues to emerge. For example, single-cell analysis identified a predominantly mesenchymal/CSC program in early-stage TNBC micro-metastases, in contrast to late-stage metastases [154]. The findings are consistent with the idea that mesenchymal/CSC initiate metastatic outgrowth at a secondary site, followed by differentiation. Single cell RNA sequencing (scRNA-seq) confirmed that EMT in primary tumors proceeds through distinct, hybrid states, ranging from completely epithelial to completely mesenchymal [155]. The epithelial-mesenchymal hybrids, which harbor the greatest level of phenotypic plasticity, are more efficient at intravasating, surviving in circulation, extravasating to the lungs, and forming metastases [175]. Acute exposure to Adriamycin or Taxanes drives the adaptive emergence of therapy-resistant, CD44^HIGH^ CSC in both breast tumor explants and breast cancer cell lines [176]. scRNA-seq determined that chemo-resistant cells activate an EMT program, which was not evident before treatment [177]. In mouse models, the ability to undergo EMT is important for therapeutic resistance [178,179]. These findings suggest that potent mesenchymal/CSC programming has significant consequences that allow cancer cells to adapt to chemotherapy and survive, ultimately contributing to therapy failure.

Indeed, STAT3 has also been implicated in acquired therapy resistance in many cancers including NSCLC, GBM, PDAC, melanoma, and breast cancer [107,180,181,182,183,184,185]. Elevated STAT3 phosphorylation in ovarian cancer is associated with paclitaxel resistance and increased tumor cell invasion post-therapy, consistent with the elevated expression of mesenchymal/CSC genes and increased tumor initiating potential [176,186,187]. In Trastuzumab-resistant HER2+ breast cancer, STAT3 feed-forward loops generate a TME rich with STAT3-activating cytokines that promote and maintain the mesenchymal/CSC phenotypes. Inhibition of the STAT3 feed-forward activation diminished tumor growth and metastasis and resensitized cells to therapy [188]. In cancer cells driven by diverse receptor tyrosine kinases (RTKs) (EGFR, HER2, ALK, and MET), MEK inhibitors drive feedback activation of STAT3 through FGFR and JAKs, resulting in therapy failure [189]. Likewise, in NSCLC cells resistant to molecularly-targeted therapies (EGFR-TKI, ALK inhibitor Crizotinib, and MEK inhibitor Selumetinib), OSM/JAK1/STAT3–signaling protects cells from targeted drug-induced apoptosis [190]. JAK/STAT3 signaling also interacts with numerous other growth, proliferative, and survival pathways, such as PI3K/Akt, MAPK, NF-kB, Notch, Wnt/β-Catenin, and TGF-β, among others [191,192,193,194,195,196]. Tamoxifen-resistant breast cancers have elevated STAT3 and Notch4 expression associated with metastasis and tumorigenicity. Interestingly, inhibition of Notch4 was able to reduce the phosphorylation of STAT3 and suppress metastasis, suggesting STAT3 and Notch4 cooperate to promote therapy resistance [197]. In addition, a STAT3/NF-kB complex promotes cisplatin resistance in malignant mesothelioma, and targeted inhibition of this complex inhibits the growth of refractory tumors [198]. Such evidence suggests STAT3 may not be solely responsible for therapy failure. Instead, STAT3-mediated cross-talk with additional signaling effectors may coordinate resistance [140,199,200,201]. Nonetheless, the common theme appears to be a STAT3-activated, mesenchymal/CSC program.

## 5. A STAT3-Generated Pro-Metastatic Immune Microenvironment

An important and rapidly emerging area of research is the impact of the immune microenvironment on metastasis and therapy failure. Beyond its impact on tumor cells, STAT3 has an important role in restricting immune cell functions and producing immunosuppressive factors. STAT3-induced cytokines are important mediators of the crosstalk between tumor cells, tumor-associated macrophages (TAMs), and tumor-associated neutrophils (TANs), which are responsible for generating a pro-metastatic and pro-angiogenic TME [202]. In the TME, macrophages and neutrophils are exposed to a host of STAT3-induced cytokines, such as IL-4, IL-6, IL-10, IL-13, VEGFA, and TGF-β that drive their polarization to a pro-tumorigenic M2 (macrophages) or N2 (neutrophils) state [203,204]. Conversely, robust STAT3 signaling activity in M2-TAMs and N2-TANs correlates with the production of factors able to drive cancer cell EMT (OSM, IL-6, TGF-β) and angiogenesis (VEGF, TGF-β, PDGF, and FGF) [205,206,207,208].

Importantly, because of their localization at the invasive edge of tumors, the contribution of M2-TAMs and N2-TANs to metastasis is increasingly being recognized. For example, TAMs have been demonstrated to form simultaneous physical contacts with tumor cells and endothelial cells that result in the formation of invadopodia, which assist cancer cells in transendothelial migration and escape into the circulatory system. These sites of cancer cell intravasation are called “tumor microenvironment of metastasis (TMEM)” and have been validated as prognostic markers of metastasis. Chemotherapy increases TMEM in breast cancer patients, thereby potentially facilitating metastasis [209,210]. STAT3 inhibition in TAM populations re-sensitizes breast cancer cells to paclitaxel, further suggesting tumor and TAM crosstalk is an essential component in STAT3-driven therapy resistance [205,211]. TANs, on the other hand, were found to function as circulatory escorts of CTC and promote their proliferation, survival, and seeding of secondary sites [212,213,214]. Importantly, OSM and IL-6 were two of the 4 most frequent cytokines secreted by neutrophils found clustered with CTC. These cytokines interact with OSMR and IL-6R, which are expressed on cancer cells [212]. The neutrophil/CTC cross-talk promotes the cell cycle progression in CTC, thereby expanding their metastatic potential.

Following the classical events of the metastatic cascade, after a tumor cell intravasates into circulation, it extravasates to establish secondary sites. An emerging concept in the metastatic cascade is the role of myeloid cells- such as basophils, neutrophils, eosinophils, monocytes, and macrophages- in the establishment of a pre-metastatic niche [215]. Although the exact TME components required for colonization of secondary sites remain a mystery, the activation of a STAT3-sphingosine 1-phosphate receptor-1 (S1PR1) axis in tumor cells, and secretion of IL-6 and IL-10, was observed to persistently activate STAT3 at distant, pre-metastatic sites. At these pre-metastatic sites, persistent STAT3 phosphorylation was associated with myeloid cell migration from the primary tumor to the secondary site. Subsequent targeting of STAT3 in myeloid cells disrupted metastatic tumor outgrowth, suggesting STAT3 plays an integral role in priming distant metastatic sites for tumor cell outgrowth [216].

## 6. STAT5 in Cancer Cells

As previously mentioned, cells produce two different STAT5 proteins, STAT5A and STAT5B, which share greater than 90% sequence homology [217]. However, evidence suggests STAT5A and STAT5B play different functional roles in normal and cancer cell systems. Genetic deletion of STAT5 in pure mouse backgrounds are embryonic lethal, due to the essential roles of STAT5 in erythropoiesis and iron metabolism [218,219,220]. While the distinct roles of STAT5A and STAT5B remain understudied, data from mammary gland-specific knockouts suggest that STAT5A is required for lactogenesis in the mammary gland, while STAT5B is imperative for mammary gland differentiation and development [221]. The function of STAT5A and STAT5B appear to be cell-specific. They can have either synergistic or opposing effects, such as in memory B-cell differentiation, which may be due to (i) the formation of STAT5A/STAT5B homo- or hetero-dimers, and/or (ii) differences in nuclear shuttling mechanisms [222,223,224]. Furthermore, genetic tuning models depleting STAT5A and/or STAT5B have demonstrated a critical role for STAT5 in the accumulation and development of innate lymphoid cells, such as NK cells [225,226,227].

Given its role in lymphoid cell development and differentiation, it is not surprising that STAT5 activity contributes to hematologic malignancies. Deletion of STAT5 prevents transformation by the Abl oncogene, thereby preventing leukemia development [228]. Genetic and pharmacologic inhibition of STAT5 activity decreases expression of apoptosis inhibitors MCL1 and BCL2 and inhibits leukemogenesis of BCR-ABL1+ acute lymphoblastic leukemia (ALL), both in cell lines and newly diagnosed and relapsed/TKI-resistant ALL patients [229]. Likewise, a new, STAT5 inhibitor suppressed the proliferation of human acute myeloid leukemia (AML) cell lines and primary FLT3-ITD+ AML patient cells [74]. Combined inhibition of STAT3 and STAT5 by shRNAs also suppressed growth in chronic myeloid leukemia, suggesting that combinatorial suppression of STAT3 and STAT5 may be efficacious in treating hematological malignancies [230].

STAT5 activation has also been implicated in the progression of solid tumor malignancies. Deletion of STAT5 in the mammary gland, hepatocytes, and prostate cells delays the development of mammary, liver, and prostate cancer [32,231,232]. Like STAT3, experimental evidence implicates STAT5B as a driver of tumorigenesis, as it can drive EMT and increased invasiveness in hepatocellular carcinoma (HCC) [233]. Moreover, in mammary epithelial cells, thymocytes, and epithelial prostate cancer cells, persistent activation of STAT5 is sufficient to drive transformation [234,235,236]. Furthermore, the function of STAT5 in solid tumors extends beyond oncogenesis as evidence has emerged that STAT5 signaling can induce a metastatic cascade. For example, STAT5 inhibition in colorectal cancer induces G1 cell cycle arrest and reduces cancer cell migration, demonstrating the role of STAT5 in proliferation and metastasis [237]. Additional studies demonstrate that STAT5A/B signaling in prostate cancer and squamous cell carcinoma of the head and neck drive EMT programming, which results in enhanced cell migration, invasion, and formation of metastases [238,239].

An increasing number of reports demonstrate that STAT5 drives tumorigenesis and cancer progression through cooperation with other intracellular signaling cascades and activation of additional feed-forward loops. STAT5 activates transcription of AKT1 and PI3K, and, in turn, Akt1 phosphorylates STAT5 to induce cell survival [240,241]. Furthermore, STAT5-dependent Akt restores cyclin D expression, which promotes proliferation [241]. Once cells aberrantly proliferate, apoptosis suppressors Bcl-xL and Bcl-2 are activated via persistent STAT5 signaling, driving tumor cell survival [16,234,242,243,244]. BCL-XL expression is also enhanced via formation of a transcription factor complex comprised of phosphorylated STAT5 and nuclear EGFRvIII. This transcription factor complex binds to the BCL-XL promoter to induce its transcription and promote anti-apoptotic signaling [245]. Similar to STAT3, STAT5 translocates to tumor cell mitochondria, suggesting an interaction with the mitochondrial genome to promote aerobic glycolysis (the Warburg Effect), a defining characteristic of cancer cells [246]. Collectively, this data demonstrates that STAT5 mediates crosstalk between cancer cell survival, and proliferation, and metabolism signaling pathways.

Surprisingly, STAT5 in certain model systems has been demonstrated to function in a tumor suppressive manner. Human breast cancers infrequently (~7%) show signs of STAT5 activation (compared to 40% of STAT3 activation). This elevated STAT5 activity trends with more differentiated and lower grade tumors, suggesting that STAT5 does not induce the aggressive cancer cell program initiated by STAT3, at least in breast cancer [247]. STAT5 expression in these models stabilizes E-cadherin surface marker expression and reverses the undifferentiated mesenchymal phenotype [248]. In normal human fibroblasts, aberrant activation of STAT5A induces a senescence response concurrent with accumulation of p53 and DNA damaged foci. Furthermore, knockdown DNA-repair kinase, ATM, and tumor suppressor, retinoblastoma protein did not eliminate damaged foci, providing evidence for the persistence of DNA damage in pre-malignant lesions [249]. Interestingly in HCC models, liver specific STAT5 knockout results in tumor formation through the enhanced activation of TGFβ/STAT3 signaling [20]. Physiologically, STAT proteins have been identified as drivers of erythropoiesis. STAT5A/B double knockout mice in a mixed genetic background (Sv129 x C57Bl/6) results in mild hematopoietic phenotypes, due to compensatory activation and enhanced DNA-binding of STAT1/3 [218].

## 7. STAT5 in T_reg_-Associated Immunosuppression

In addition to its impact on cancer cells, activated STAT5 dampens anti-tumor immune function. This immunosuppressive function is largely driven by CD4+/CD25+ T_regs_, a subset of T cells that contribute to tumor progression and metastasis [250] and correlates with poor patient prognosis [251,252]. Sustained STAT5 phosphorylation in progenitor T cells induces the differentiation to a T_reg_ population that, in turn, significantly diminishes the function of cytotoxic and helper T cells [253,254,255]. Experimental depletion of T_regs_ from the TME results in enhanced infiltration of mature CD4+ and CD8+ T cells into the tumor, leading to tumor rejection [256,257]. Furthermore, STAT5-mediated T_reg_ expansion increases IL-10, IL-4, and IL-13, which skew TAMs to an M2 immunosuppressive phenotype. M2 macrophages are immunosuppressive because they release elevated levels of IL-10 and transforming growth factor-β (TGF-β) and restrict secretion of immune stimulatory cytokines via NF-kB repression [258,259]. STAT5-induced T_regs_ and M2 macrophage populations secrete VEGF-A, which, along with TGF-β, promotes angiogenesis [255,260,261].

Tumor clearance mechanisms are also suppressed by T_reg_ expansion through impediment of B cell development and maturation [262]. STAT5-activated T_regs_ result in a decrease in follicular helper T (T_fh_) cell populations via Blimp-1, which severely hampers germinal center formation in lymph nodes [263]. This reduction in germinal centers diminishes the number of B cells that can be recruited and primed to aid in an anti-tumor immune response. Interestingly, the differentiation and self-renewal of memory B cells are respectively influenced by STAT5A-mediated repression and STAT5B-mediated induction of BCL-6. Immunosuppression driven by the expansion of T_regs,_ relies on STAT5-mediated alteration in T-cell metabolism. Mature effector T cells preferentially undergo glycolysis and require a de novo fatty acid synthesis reliant on acetyl-coA carboxylase 1 whereas T_regs_ undergo lipid-oxidation and readily synthesize fatty acids because of a structural reconfiguration of mitochondrial cristae [264,265,266]. Accumulation of intracellular lipids impairs autophagy, providing a mechanism for T_reg_-mediated immunosuppression [267,268]. Just as STAT5 function in the mitochondria impacts tumor cell functions, as described earlier, mitochondrial STAT5 activation drives metabolic shifts in the immune compartment, inducing an expansion of T_regs_.

However, while the suppression of T_regs_ increases tumor immunity, provided STAT5 remains functional, the overall impact of suppressing STAT5 signaling in the remaining immune cells of the TME remains open to debate. Mouse models deficient in STAT5 have depleted CD8+ T cell, NK cell, and T_reg_ populations, suggesting STAT5 plays an integral role in the proper development of multiple immune cell types [269]. STAT5 signaling contributes to the differentiation of naive CD4+ T cells into CD8+ T cells, T_h_1, T_h_2, T_h_9, T_h_GM, and T_regs_, while inhibition of STAT5 is required for the generation of T_h_17 and T_fh_ cells [270,271]. Importantly, STAT5 is heavily involved in the development, survival, and lytic function of NK cells. Knock-out or suppression of STAT5 in NK populations sparked the secretion of VEGF-A, a growth factor that supports tumor-associated angiogenesis in melanoma and leukemia models [269,272]. Taken together, these findings suggest that targeting STAT5 may threaten the integrity of anti-tumor immune functions and drive worse outcome in patients [269,272].

## 8. Targeting STAT Activity

**Figure 2:**
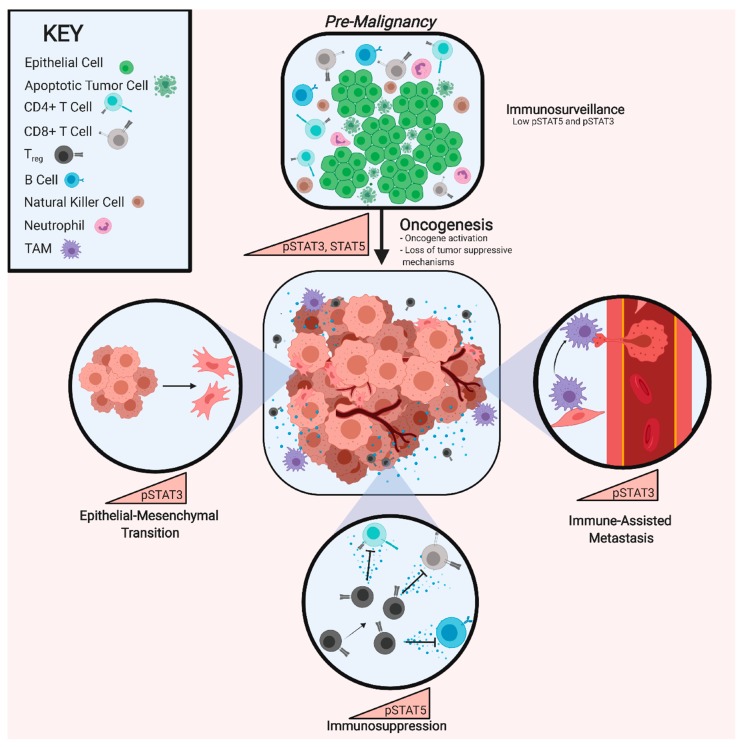
Biological Impact of STAT3 and STAT5 Activation. Pre-malignant cell populations, in which apoptotic signaling and immunosurveillance are functional, exhibit low levels of phosphorylated STAT3 (pSTAT3) and STAT5 (pSTAT5). Elevated activity of pSTAT3 and/or pSTAT5 accompanies tumorigenesis, leading to the inhibition of apoptotic pathways and repression of immune cell recognition of a burgeoning tumor. pSTAT3 is utilized by malignant cell populations to drive epithelial-mesenchymal transition (EMT) and by tumor-associated macrophages (TAMs) and tumor-associated neutrophils (TANs) to drive metastasis. pSTAT5 activity in progenitor T-cells drives expansion of a Treg population that then secretes factors that inhibit the function of CD4+ and CD8+ T-cells as well as B-cells.

Given the roles of STAT3 and STAT5 in tumor progression and immunosuppression discussed above (Figure 2), multiple methods of inhibiting their activity are being pursued. Successful inhibition of STAT3 would prevent the acquisition of, and potentially revert, a mesenchymal/CSC program, making cancer cells less invasive and more sensitive to therapy. Moreover, STAT3 inhibition would help activate anti-tumor immunity by reducing immunosuppressive factors and increasing the infiltration of immune cells into the TME. Likewise, suppressing STAT5 in cancer cells, particularly leukemia, halts proliferation and induces apoptosis, suggesting that STAT5 may be a valuable therapeutic target [74]. However, due to STAT5′s controversial roles in tumor progression and immune cell maturation and differentiation, further studies are required to elucidate the effects of targeting STAT5 in cancer patients. [273]. Generally speaking, the majority of small molecule inhibitors are designed to have high affinity for the catalytic domain of an enzyme; the ATP binding site for example, which STAT proteins lack. Therefore, direct inhibition of STAT3 relies on disruption of binding motifs necessary for downstream signal transduction. A series of STAT3 phospho-ester SH-2 domain inhibitors have been developed, with the intent to inhibit dimerization of activated STAT3 (PY*L, S3I-2001, OPB-31121, etc.). While these approaches are promising, the compounds continue to be refined [274,275,276,277]. An alternative approach would involve targeting the upstream activator of STAT3, which could include neutralizing antibodies for specific IL-6 family cytokines, competitive antibodies hindering cytokine-receptor interactions, and JAK inhibitors.

One of the first IL-6 monoclonal antibodies generated was Tocilizumab, which inhibits IL-6 signaling by preventing IL-6 binding to both the soluble and transmembrane forms of IL-6R [278,279]. Pre-clinical studies demonstrated strong anti-tumor cell activity in multiple myeloma and therapeutic efficacy in Castelman’s disease, rheumatoid arthritis, and cytokine release syndrome, however, Tocilizumab is not currently FDA-approved for cancer treatments [280,281,282,283]. Clinical trials using Tocilizumab in combination with other monoclonal antibodies, chemotherapies, and immunotherapies to treat cancers such as HER2+ breast cancer, B-cell Non-Hodgkin Lymphoma, metastatic NSCLC, and recurrent metastatic colorectal adenocarcinoma are ongoing [284]. In addition to Tocilizumab, Siltuximab is an IL-6 specific neutralizing antibody that has emerged as another promising therapy. Pre-clinical studies support Siltuximab use in KRAS-mutant lung cancer, particularly in tumors with elevated stromal production of IL-6 [285]. Similar to Tocilizumab, Siltuximab is undergoing clinical trials for patients with malignant solid tumors in cancers with elevated stromal presence, such as ovarian, pancreatic, colorectal, head and neck, and lung neoplasms [286]. Furthermore, phase II studies of combination therapy with Siltuximab have demonstrated anti-tumor effects for patients diagnosed with metastatic prostate cancer, suggesting clinical efficacy of targeting the IL-6-STAT signaling axis with combination therapies [287].

Our lab has focused on OSM, given its potent activity at inducing numerous inflammatory cytokines that promote mesenchymal/CSC reprogramming in cancer cells and generate a pro-tumor immune microenvironment [106,288,289,290]. Neither OSM nor OSMR protein are abundantly expressed in normal tissues in the absence of inflammation, in contrast to JAK1, JAK2, and STAT3 (key OSM effectors) [109]. This finding is supported by the observation that knocking out OSM or OSMR from mice results in only mild phenotypes [291]. Moreover, OSMR has characteristics unique from other IL-6 family co-receptors, resulting in distinct signaling and biological effects [43,292,293]. For example, OSMR strongly recruits SHC, resulting in the hyper-activation of the MAPK signaling cascade [293]. Other gp130 co-receptors fail to do this, and rely solely on the gp130-mediated SHP-2 recruitment for MAPK activation, which is less robust than the gp130/OSMR heterodimer [43]. Therefore, suppressing the OSM signaling axis may have benefits in aggressive subtypes of cancer. Anti-OSM antibody (GSK315234) and OSMR-fusion protein (OR-FC) were first examined in pre-clinical studies in chronic inflammatory disease models, such as rheumatoid arthritis and inflammatory heart disease [294,295]. Antibodies suppressing OSM signaling ameliorate these pathological conditions, suggesting that OSM is a driver of these chronic hyper-inflamed states. More recently, OSM signaling was identified as a driver of inflammatory bowel disease (IBD), especially in patients who fail to respond to anti-tumor necrosis factor-α (TNF-α) antibodies. Neutralization of OSM in IBD models suppresses a cadre of inflammatory cytokines and reduces colitis severity, further supporting the OSM feed-forward loop as a critical mediator in the long-term maintenance of inflammatory signaling, a state common in the TME [112].

Another recent study investigating the effects of an anti-OSM antibody in a murine model of lupus nephritis demonstrated that OSM-driven EMT and extracellular matrix secretion, leading to renal fibrosis, could be suppressed, concomitant with the suppression of JAK/STAT3 activation [296]. More recently, a clinical grade OSM neutralizing antibody was used to treat pre-clinical models of squamous cell carcinoma. Again, OSM neutralizing antibodies suppressed the STAT3 feed forward signaling, resulting in reduced invasion and metastasis [96,297]. Disrupting cell surface receptor activation of STAT3 by inhibiting OSMR signaling was recently described in a study of an OSMR/gp130 antagonist (SN79), which prevents STAT3 phosphorylation in astrogliosis [298]. While these studies demonstrate the potential of OSM and OSMR inhibition as a therapeutic strategy, a number of ligand/receptors activate STAT3 and STAT5, as described above. Therefore, additional studies will be needed to define whether single ligand-receptor inhibitors can sufficiently impact STAT3 activation, thus suppressing tumor growth.

Currently, JAK inhibitors are the most promising inhibitors of STAT-driven phenotypes. Commonly used JAK 1/2 inhibitor, Ruxolitinib, has been shown to robustly block both STAT3 and STAT5 activation. Yet, while JAK inhibitors can reduce STAT3 activation, there is conflicting data on the impact of JAK inhibitors. Some studies find that JAK inhibition suppresses tumor growth [93], while other studies find that they ultimately enhance metastasis, likely because they also suppress the positive influence of JAK activity on other STAT proteins (including STAT5 and STAT1/2, as discussed below) [299]. In addition, side effects of JAK inhibitors may be more pronounced, as JAKs activate other pathways, such as MAPK and PI3K in normal cells as well as cancer [43].

## 9. Balancing Opposing STAT-Activated Cytokine Signaling as a Therapeutic Strategy

Though STAT proteins are grouped together based on their structural similarities and common functions as transcription factors, individual STAT proteins can have diverse functions. We have focused extensively on STAT3 and STAT5 and their described roles in cancer progression, however this is not the universal effect among all STATs. For example, Interferon-β (IFNβ) induces the phosphorylation and activation of STAT1/2, which form a complex with IRF9 to create the transcription factor complex ISGF3. IFNβ/P-ISGF3 signaling induces interferon-stimulated genes (ISG), mesenchymal-epithelial transition (MET), and the differentiation of CSC into a less aggressive epithelial, non-CSC state with reduced migratory potential and reduced tumorsphere forming capabilities [111,300,301]. Importantly, the IFNβ and OSM/STAT3 signaling pathways strongly oppose one another. OSM represses transcription of IFNβ, thereby eliminating autocrine and paracrine IFNβ-mediated activation of P-ISGF3 and repressing ISG expression in both cancer cells and immune cells [301].

In addition to the impact of IFNβ on cancer cells, increasing rationale supports developing methods for the delivery of P-ISGF3 activators (or Type I IFN-agonists more generally) directly to the TME. First, favorable responses to frontline chemotherapy correlate with robust IFN signaling in both mouse and human studies [111,302,303,304]. Elevated IFN signaling in the tumor correlates with immunologically “hot” tumors harboring elevated numbers of tumor infiltrating lymphocytes (TILs), activated immune surveillance, increased tumor antigen cross presentation, and diminished numbers of immunosuppressive cells including MDSCs and T_regs_ [111,302,305,306,307]. Second, loss of Type I IFN signaling correlates with metastasis and decreased survival. Restoration of Type I IFN signaling significantly decreases metastasis and improves survival outcome [303,304]. Third, in contrast to STAT3 activators (which promote a pro-tumorigenic M2 state), addition of type I IFN inhibits macrophage polarization to an M2 state [308,309]. Type I IFNs also induce the differentiation of neutrophils into anti-tumor N1s [310]. Fourth, administration of IFNβ prior to surgical resection significantly improves response rates to immunotherapies such as anti-PD1/anti-PDL-1 [311]. Therefore, we propose balancing pro-tumor STAT3 activation with anti-tumor STAT1/STAT2 activation as a novel therapeutic approach. This STAT3/STAT1 balancing would (i) reprogram mesenchymal/CSC to a non-CSC state, making them more susceptible to chemotherapy and (ii) enhance anti-tumor immunity, thereby facilitating immune cell-mediated tumor cell killing. Yet, while IFN treatment is currently approved to treat hematological malignancies and some solid tumors (melanoma), the high doses of IFNs needed to inhibit cancer cell proliferation or induce cell death result in side-effects that limit its effectiveness [311,312,313]. We suggest tumor targeting antibodies (or nanobodies) linked to IFNβ. Generation of an oncogenic cytokine or receptor antibody conjugated to an IFNβ first demonstrated success in limiting resistance to EGFR inhibitors in breast cancer [314]. The designed therapy sought to re-activate innate and adaptive immune components, while simultaneously targeting the oncogenic receptor EGFR [303]. IFNβ-conjugated antibodies show immense promise. They would limit toxicity by using tumor-associated receptors to target IFNs to the TME and suppress STAT3 activation, while simultaneously, activating STAT1/2 in both tumor cells and immune cells [315].

## 10. Conclusions

As discussed throughout this review, STAT3 and STAT5 have emerged as essential components involved in regulating tumor progression. Cytokines and cytokine receptors of the IL-6 family are some of the most widely recognized STAT activators and are abundantly expressed on cancer cells as well as tumor-infiltrating immune cells. The role of STAT3 in promoting molecular programs in cancer cells that induce tumor metastasis and therapy resistance mechanisms continue to emerge, as does the impact of STAT3 as a suppressor of immune cell function in the TME. These findings suggest that specifically suppressing STAT3 activation would be beneficial to patients. Targeting STAT5 in hematological malignancies is gaining traction, and the clinical successes of JAK and tyrosine kinase inhibitors in disrupting STAT3 and STAT5 activation, provide strong support for the development of direct STAT3 and STAT5 inhibitors, summarized in Table 1. Specific suppression of STAT5 in immune-suppressive T_regs_ could also prove beneficial, but STAT5 is essential for many other immune cells as well. Thus, systemic suppression of STAT5 activity could undermine tumor immunity and promote tumor progression, as recently reported for STAT5 knock-out from NK cells [272]. However, reducing the aberrant activation of STAT5 without complete ablation may have therapeutic efficacy upon combination with other vulnerabilities. We conclude that targeting STAT3, either directly by disrupting STAT3-homodimer formation or indirectly by suppressing the activation of receptors responsible for persistent STAT3 phosphorylation, would reverse the cellular programs driving metastasis and therapy failure. Furthermore, by activating STAT1/2 within TME cells, the programs that prevent metastasis and enhance therapeutic efficacy could be re-engaged. This STAT balancing would improve outcomes for patients, particularly those with aggressive cancers that may currently have limited therapeutic options.

## Figures and Tables

**Table 1:** Summary of the Roles of STAT3 and STAT5 in Cancer and Strategies for their Inhibition.

STAT-Family Protein	pYSTAT3/5-Activating Cytokines and Growth Factors	Normal Immune Function	Pro-Tumorigenic Genes Activated	Pro-Tumorigenic Effects	Therapies and Drugs in Development
STAT3	**Growth Factors:** EGFs, FGFs, HGFs, Leptin (REF), PDGFs **Lymphoid Cytokines:** IL-4, IL-13 **Myeloid Cytokines:** G-CSF, IL-10 **Ubiquitous Cytokines:** CNTF, LIF, IL-6, OSM	Development of Mature Neutrophils [58] Neutrophil Mobilization from Bone Marrow [59]	**Apoptosis:** BCL2, BCL-XL, MCL1, PIM1 **Cell Cycle:** Cyclin A, Cyclin D1, CDC25A **EMT:** ZEB1, SNAI1, TWIST, CDH2, Vimentin **Migration/Invasion:** MMP2, MMP9, VEGFA **Proliferation:** cMYC, NOTCH4 **Stem Cell:** SOX2, NANOG, OCT4 **Tumor Suppressor:** p53	Transformation Activation of Anti-Apoptotic Proteins EMT M2 Polarization N2 Polarization Enhanced Metastasis Therapy Resistance	Imatinib (JAK1/2) Ruxolitinib (JAK1/2) Tofacitinib (JAK1/2) *Siltuximab* (IL-6) *Tocilizumab* (IL-6R) *GSK315234* (OSM) *OS-FC* (OSMR) PY*L (SH2 Domain) *S3I*-*2001* (SH2 Domain) *SN79* (Sigma Receptor) IFNβ
STAT5	**Growth Factors:** EGFs, FGFs, FLT3L, Leptin [60], PDGFs, Prolactin, SCF **Lymphoid Cytokines:** IL-2, IL-4, IL-7 [61], IL-9 [62], IL-13, IL-15, Thymic Stromal Lymphopoietin [63] **Myeloid Cytokines:** Erythropoietin [64], G-CSF, GM-CSF, IL-3, IL-5, IL-10, Thrombopoietin [65] **Ubiquitous Cytokines:** IL-21 [66], IL-31 [67], OSM	Differentiation, Survival, and Lineage Expansion of NK and NKT Cells Dendritic Cell Function [68] T Cell Differentiation and Expansion Effector Memory CD8+ T Cell-Mediated Cancer Cell Clearance [69] Myeloid Cell Differentiation and Survival [70]	**Apoptosis:** BCL-2, BCL-XL, MCL1, PIM1, Survivin **Cell Cycle:** Cyclin D1, Cyclin E **Immune Function:** PRDM1, BCL-6 **Migration/Invasion:** MMP2, MMP9, VEGFA **Proliferation:** AKT1, cMYC, PI3K **Tumor Suppressor:** p53	Transformation Activation of Anti-Apoptotic Proteins T_reg_ Population Expansion M2 Polarization [71] Megakaryopoeisis [72] Erythropoeisis [73]	Imatinib (JAK1/2) Ruxolitinib (JAK1/2) Tofacitinib (JAK1/2) *Siltuximab* (IL-6) *Tocilizumab* (IL-6R) *GSK315234* (OSM) *OS-FC* (OSMR) *AC-4-130* (SH2 Domain) [74] *BP-1-107* (SH2 Domain) [75] *BP-1-108* (SH2 Domain) [75] *Pomstafib-2* (SH2 Domain) [76] *SF-1-087* (SH2 Domain) [75] *SF-1-088* (SH2 Domain) [75] IFNβ

Therapies included in the far right column are listed by their respective name followed by their biological target in parentheses. Italicized font denotes therapies that are not currently approved by the FDA as cancer therapies.
